# SEMAC + VAT for Suppression of Artifacts Induced by Dental-Implant-Supported Restorations in Magnetic Resonance Imaging

**DOI:** 10.3390/jcm12031117

**Published:** 2023-01-31

**Authors:** Lauren Bohner, Marcel Hanisch, Hian Parize, Newton Sesma, Johannes Kleinheinz, Norbert Meier

**Affiliations:** 1 Department of Cranio-Maxillofacial Surgery, University Hospital Muenster, 48149 Muenster, Germany; 2 Department of Prosthodontics, School of Dentistry, University of São Paulo, São Paulo 05508-220, Brazil; 3 University Hospital Muenster, 48149 Muenster, Germany

**Keywords:** dental implants, magnetic resonance imaging, artifacts

## Abstract

The purpose of this study was to assess the feasibility of SEMAC + VAT to reduce artifacts induced by dental implant-supported restorations, such as its impact on the image quality. Dental-implant supported restorations were installed in a dry mandible. Magnetic resonance scans were acquired on a 3-Tesla MRI system. Artifact suppression (SEMAC + VAT) was applied with different intensity modes (weak, moderate, strong). Artifacts assessment was performed by measuring the mandible volume increase in MRI images prior (reference dataset) and after installation of dental implant-supported prosthesis. Image quality was assessed by two examiners using a five-point scale. Inter-examiner concordance and correlation analysis was performed with Cronbach’s alpha and Spearman’s test with a significance level at *p* = 0.05. Mandible volume increased by 60.23% when no artifact suppression method was used. By applying SEMAC + VAT, the volume increase ranged from 17.13% (strong mode) to 32.77% (weak mode). Visualization of mandibular bone was positively correlated with SEMAC intensity degree. SEMAC + VAT reduced MRI artifacts caused by dental-implant supported restorations. A stronger suppression mode improved visualization of mandibular bone in detriment of the scanning time.

## 1. Introduction

Magnetic resonance imaging (MRI) is widely used for medical diagnosis of head and neck area. Recently, this method has shown its potential for intraoral and dental assessment [1,2,3,4,5,6,7]. However, despite the high quality of MRI, unintended effects known as artifacts can hamper image quality requiring closer attention to avoid diagnostic errors [8,9,10].

Artifacts arise either intrinsically from the MRI system or from its interaction with the scanned body. Among all types of unintended effects, susceptibility artifacts produced by dental materials play an important role on MRI of head and neck area. Dental materials create magnetic field distortions leading to variations across neighboring tissues producing signal loss and spatial misplacing of the signal. Additionally eddy currents caused by gradient fields in metal implants may unfold even more severe B0 distortions [11].

A proper adjustment of MRI parameters can be effective to reduce these artifacts. For this purpose, different strategies like lower strength fields, spin echo sequences, changing slice thickness or orienting the implant parallel to the magnetic field may be employed [11,12,13]. Moreover, artifact reduction techniques, such as “Slice Encoding for Metal Artifact Correction” (SEMAC) and View Angle Titling (VAT), were developed to adjust the signal loss caused by metallic materials, and have shown promising results for medical applications [14,15,16,17].

VAT compensates in-plane geometric distortions by means of a slice-selection gradient during signal readout, which corrects trueness of pixel position within the image plane. However, artifacts correction may result in geometric-through-plane distortion and blurring effect. Since VAT alone is not able to correct through-plane distortions, it is therefore typically applied to reduce less severe in-plane artifacts. In order to correct through-plane distortions, SEMAC employs additional z-phase encoding steps to restore through-plane distortions. Both methods may be used simultaneously to improve image quality [11,14,15].

Although the combination SEMAC + VAT allows assessment of anatomical structures surrounding metallic implants [14,15], it is associated with prolonged acquisition times. This in turn allows for more motion artifacts caused by breathing and swallowing, which is even more detrimental to the image quality than susceptibility artifacts alone. To address this issue, a weaker degree of artifact suppression is a widely accepted practice for reducing scanning time [11,18].

The scanning protocol should balance out the most effective artifact reduction achieved and clinically acceptable acquisition time. However, there is a lack of prior studies showing the application of these artifact reduction techniques to evaluate intraoral tissues. Thus, the purpose of this study was to assess the feasibility of SEMAC + VAT to reduce artifacts induced by dental implant-supported restorations compromising MRI image quality. The clinical applicability of this protocol took both degree of SEMAC + VAT and scanning time into consideration.

## 2. Materials and Methods

### 2.1. Study Design

Magnetic resonance (MR) scans were acquired prior and after embedding the dental-implant supported restorations in a dehydrated mandible. The pre-implant MRI represented a reference dataset not exhibiting susceptibility and eddy current artefacts. SEMAC + VAT prototype were assessed with different degrees of artifact suppression (weak, moderate, strong). Artifacts dimension and image quality resultant from each scan mode were evaluated.

### 2.2. Specimen Preparation

**Figure 1:**
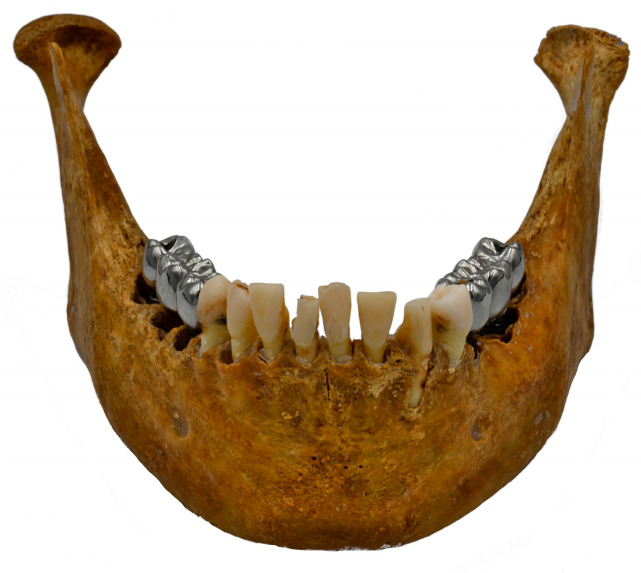
Partial dentate dry mandible containing a three-unit Co-Cr dental prosthesis supported by two titanium dental implants in each semi-arch.

A dry mandible with bilateral posterior edentulism was used in this study (ethical approval Nr. 2.253.943). Two dental implants (Bone Level Titan SLA, Straumann, Basel, Switzerland) were installed on each semi-arch (sites 35, 37, 45, 47), and a three-unit metallic restoration was manufactured (Figure 1). Composition of dental implants and prosthetic restoration are described in Table 1.

### 2.3. Magnetic Resonance Imaging

The dry mandible was inserted in a plastic tapware filled with a solution containing water, detergent solution, and contrast agent (Gadolinium 0.5 mmol/mL) to simulate the soft tissue. Scans were performed on a 3-Tesla MRI system (Philips 3.0 T Achieva) using a head coil. A coronal proton-density-weighted (PDW) turbo-spin-echo (TSE) sequence was used (Table 2).

### 2.4. Artifact Assessment

Artifact segmentation and three-dimensional (3D) reconstruction were performed using the software Imalytics Pre-Clinical (Gremse-IT GmbH, Aachen, Germany). First, mean signal intensity values of mandible and its outer surface were calculated for the reference dataset, which was free of artifacts. These mean values determined the threshold used for further segmentations, and signal intensity loss under the threshold values was considered as artifacts.

**Figure 2:**
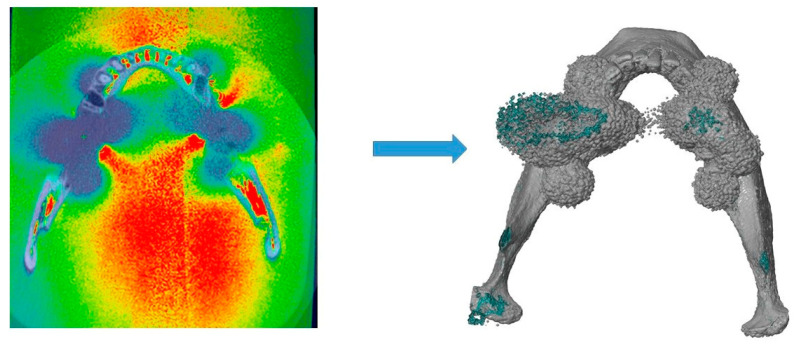
Artifacts segmentation and 3D rendering. Dark areas represent artifacts, which were converted in a three-dimensional model combined with the mandible. Mandibular volume increase was assessed to determine the artifacts dimension.

Hence, segmentation of mandibular structure and artifacts was defined based on the pre-determined threshold values. Using a combination of automatic and manual tools, both segmentations (artifacts and mandible) were combined, generating a unique set, from which a 3D geometric model was rendered and exported as a standard tessellation language (STL) data (Figure 2). Artifacts dimension was determined based on the mandible volume increase in comparison to the reference dataset. Model volume was automatically calculated using the software GOM Inspect (GOM Metrology GmbH).

### 2.5. Image Quality

Regions of interest (ROIs) measuring 4 × 4 cm were determined in axial, coronal, and sagittal planes at five sites: anterior mandible, and bilaterally at premolar and molar sites. In total, 35 images were evaluated by two blinded examiners (HP and MH) with experience in Implantology (3 and 12 years). Presence of artifacts (yes/no) and visibility of anatomical structures (cortical bone, trabecular bone, teeth and inferior alveolar nerve) were assessed. A five-point scale was used: (1) visualization not possible; (2) visualization lower than 25%; (3) visualization between 25–50%; (4) visualization between 50–75%; (5) visualization between 75–100%.

### 2.6. Statistical Analysis

Statistical analysis was performed using the software SPSS 28.0 (IBM SPSS Statistics, Ehningen, Germany). For statistical purposes, the five-point scale was clustered in three categories as follows: visualization not possible (Score 1); visualization partially possible (Scores 2–4); visualization possible (Score 5). The number of images distributed in each score were presented as percentage (%). Inter-examiner concordance was assessed using Cronbach’s alpha. Correlation between degree of artifact suppression, presence of artifacts, and visualization of anatomical structures was assessed by Spearman’s test with a significance level at *p* = 0.05.

## 3. Results

### 3.1. Artifact Assessment

**Figure 3:**
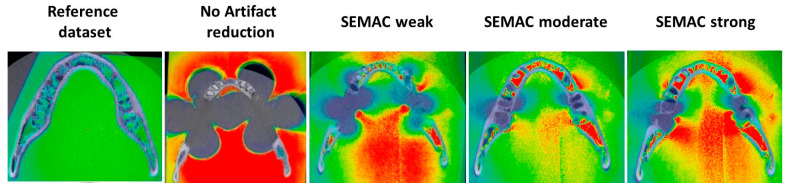
Illustrative comparison among different MRI acquisition modes.

**Figure 4:**
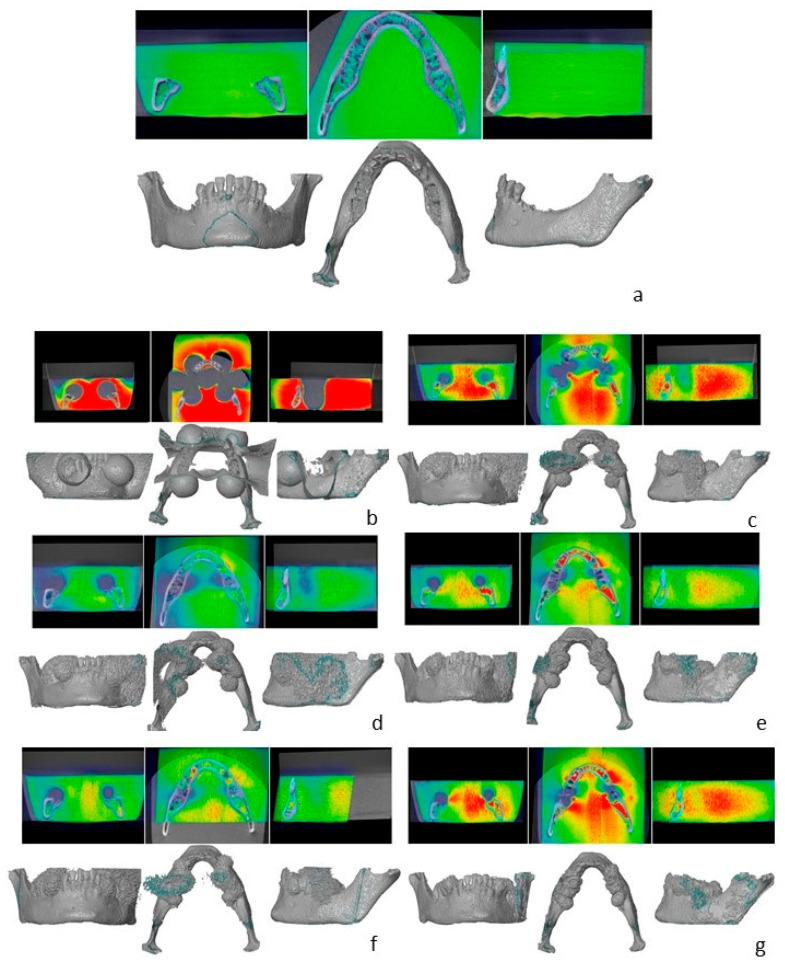
Overview of three-dimensional reconstruction in coronal, axial, and sagittal overviews. (**a**) Reference dataset. (**b**) no SEMAC. (**c**) SEMAC weak. (**d**) SEMAC weak, FID reduction. (**e**) SEMAC moderate. (**f**) SEMAC moderate, transverse direction. (**g**) SEMAC strong.

Images without metal artifact correction showed severe artifacts, which increased mandibular volume by more than 50% in comparison to the reference dataset. Artifacts were significantly reduced when SEMAC + VAT was applied. As shown in Table 2 and Table 3, SEMAC intensity degree was inversely proportional to the artifact volume and directly proportional to the scanning time. Visual assessment of artifacts is shown in Figure 3 and Figure 4.

### 3.2. Image Quality

Inter-examiner concordance was moderate to excellent (0.73–0.98), being lower for axial images. The degree of artifact suppression was positively correlated with the visibility of mandibular bone. Visualization of cortical bone increased from 20% to 100% when applying metal artifact reduction, which was positively correlated with SEMAC intensity degree in sagittal (c = 0.54; *p* < 0.01) and axial (c = 0.31; *p* = 0.03) images. Likewise, trabecular bone visualization increased from 60% to 80–100% when using metal artifact reduction. Bone assessment was negatively correlated with artifact intensity for sagittal images. Teeth and nerve visualization increased by 40% when applying SEMAC, showing no statistical correlation to SEMAC intensity.

## 4. Discussion

Despite the high potential of MRI in Dentistry [19], susceptibility artifacts produced by metallic materials can hamper image quality and prevent medical diagnosis [20]. The aim of this study was to evaluate whether these artifacts could be reduced using different degrees of artifact suppression within a clinically acceptable scan time. In summary, artifacts produced by dental-implant supported restorations were substantially reduced by 43% when using SEMAC + VAT techniques. These findings are in accordance with a previous study assessing SEMAC + VAT, which showed that artifacts caused by stainless steel and titanium implants can be reduced by 41% to 78%, especially when combining both techniques [14].

Previous studies have already shown the feasibility of SEMAC and VAT to reduce metal-induced artifacts [18,21,22,23]. However, some scanning protocols are related with a longer scanning time, and their application for MRI intraoral assessment is questionable, since this area is susceptible to motion artifacts caused by swallowing and breathing [15]. In this study, MRI parameters were adjusted to provide the maximal artifacts reduction achieved in a clinically acceptable scanning time. Several parameters were taken into consideration, such as the bandwidth (i.e., field of view), number of slices, and slice-encodings. By increasing slice encoding in transverse direction, the number of slices increased from 54 to 80, resulting in a five minutes longer scan time. Thus, despite the improved image quality, the longer scanning time may prevent its clinical use [18].

The findings of this study showed that a higher degree of artifact suppression resulted in a higher visibility of bone structure. However, considering the relation between artifacts reduction and scanning time, both weak and moderate modes should be clinically acceptable options to reduce dental-implant induced artifacts. Considering all degrees of artifacts suppression, the highest signal loss was limited to the suprastructure, enabling delimitation and recognition of bone structure. Thus, the findings of this study suggest that the evaluated protocols allow to visualize peri-implant bone.

Besides MRI parameters, implant and restorations features also influence the appearance and severity of metal-induced artifacts [24]. Based on the interaction between magnetic field and substance being scanned, biological tissues and materials present different degrees of magnetic susceptibility. They can either show a negative susceptibility and be repelled by the magnetic field (diamagnetic substances) or present a positive susceptibility and be attracted to it (paramagnetic, ferromagnetic substances). For instance, biological tissues are mostly diamagnetic, whereas Titanium and Cobalt are classified as paramagnetic and ferromagnetic alloys, respectively. Hence, because of the different magnetic properties, metallic materials, according to their alloys, can weaken or enhance magnetic fields, producing different degrees of artifacts [25,26]. Titanium alloy, for instance, produces moderate artifacts, whereas Co-Cr restoration are more susceptible to causing severe artifacts [11].

Although several studies claimed that titanium implants alone may lead to the appearance of localized artifacts, these findings are mostly related with single dental implants. However, dental implant-supported fixed prosthesis results in extended signal loss, hampering the visibility of intraoral structures [21]. Nonetheless, these findings should be carefully interpreted. Since different treatment options are available to replace missing teeth, artifacts appearance and severity may vary to each clinical scenario. Thus, these outcomes cannot be generalized, and each clinical case should be individually considered. In addition, assessment of artifacts caused by each dental material separately, i.e., dental implants and restorations, was not conducted in this study. The need to remove screwed-restorations prior to MRI should be considered in further investigations.

A limitation of this study is that clinical aspects, as patient’s motion in the presence of living tissues, were not considered in this study. Furthermore, artifact assessment was limited to the signal loss around the mandible, and slice distortions away from the mandibular structure were not evaluated. Likewise, qualitative assessment was limited to the recognition of anatomical structures, and distortion on surrounding tissues caused by different MRI sequences was not assessed. Further studies are required to achieve a faster scanning time, and assess its feasibility in a clinical scenario.

## 5. Conclusions

SEMAC + VAT reduced MRI artifacts caused by dental-implant supported restorations. A stronger degree of suppression mode improved visualization of mandibular bone to the detriment of the scanning time.

## Figures and Tables

**Table 1:** Specifications of dental materials used in this study.

Dental Implants		
**Specifications**	**Material/Alloy**	**Brand**
sites 35, 45: 2 dental implants - diameter × length: 4.1 × 12 mm sites 37, 47: 2 dental implants - diameter × length: 4.1 × 8 mm	Titanium Grade 4 (Ti, O ≤ 0.4%, Fe 0.25–0.5%, N ≤ 0.05%, C ≤ 0.10%, H ≤ 0.012%)	Bone level Titanium SLA, Straumann
**Restorations**		
**Specifications**	**Material/Alloy**	**Brand**
sites 35–37, sites 45–47: three-unit metallic restoration	Abutment	Variobase, Straumann
	Bridge: 63% Co, 29% Cr, 6% Mo, < 1% Fe, Mn, Nb, Si	Bridge: Colado CAD CoCr4, Ivoclar

**Table 2:** MRI-sequences tested for this study. Turbo spin echo (TSE) in 3D and parallel (Sense) in first level SAR acquisition sometimes using compressed sensing (CS); SEMAC—slice encoding for metal artifact correction; VAT—view angle tilting.

Scan	Image Parameters	Scanning Time
**Reference dataset** 3D TSE factor 16, Sense 2,3, no CS, 100 slices, first level SAR	Image voxel size 0.15 × 0.15 × 0.5 mm, FOV 140 × 100 mm	6 min
**No SEMAC no VAT** 3D TSE factor 16, no Sense, no CS, 200 slices, first level SAR	Image voxel size 0.15 × 0.15 × 0.25 mm	8 min
**SEMAC + VAT weak**	Image voxel size 0.25 × 0.25 × 1.0 mm	5 min
**SEMAC + VAT weak** strong FID reduction and TSE 8	Image voxel size 0.25 × 0.25 × 1.0 mm	5 min
**SEMAC + VAT medium** 54 slices	Image voxel size 0.25 × 0.25 × 1.0 mm	10 min
**SEMAC + VAT medium** Slice encoding in transverse direction, 80 slices	Image voxel size 0.25 × 0.25 × 1.0 mm, FOV 160 × 80 mm	15 min
**SEMAC + VAT strong**	Image voxel size 0.25 × 0.25 × 1.0 mm	23 min

**Table 3:** Mandible volume (cm^3^) and volume increase (%) in comparison to the reference dataset.

	Volume (cm^3^)	Volume Increase (%)
Reference dataset	3.654, 85	-
No	7.348, 17	60, 23
SEMAC + VAT weak	5.856, 21	31, 47
SEMAC + VAT weak	4.858, 25	32, 77
SEMAC + VAT medium	4.805, 39	19, 76
SEMAC + VAT medium	4.377, 16	19, 33
SEMAC + VAT strong	4.311, 21	17, 13

## Data Availability

Data available on request from the authors.
